# False News of a Cannabis Cancer Cure

**DOI:** 10.7759/cureus.3918

**Published:** 2019-01-19

**Authors:** Siyu Shi, Arthur R Brant, Aaron Sabolch, Erqi Pollom

**Affiliations:** 1 Radiation Oncology, Stanford University, Stanford, USA; 2 Radiation Oncology, Kaiser Permanente Interstate Radiation Oncology Center, Portland, USA

**Keywords:** cannabis, cancer care, alternative medicine, social media, online health information, google trends, false news

## Abstract

Background

There is increasing concern among healthcare communities about the misinformation online about using cannabis to cure cancer. We have characterized this online interest in using cannabis as a cancer treatment and the propagation of this information on social media.

Materials & methods

We compared search activity over time for cannabis and cancer versus standard cancer therapies using Google Trends’ relative search volume (RSV) tool and determined the impact of cannabis legalization. We classified news on social media about cannabis use in cancer as false, accurate, or irrelevant. We evaluated the cannabis-related social media activities of cancer organizations.

Results

The online search volume for cannabis and cancer increased at 10 times the rate of standard therapies (RSV 0.10/month versus 0.01/month, p<0.001), more so in states where medical or recreational cannabis is legal. The use of cannabis as a cancer cure represented the largest category (23.5%) of social media content on alternative cancer treatments. The top false news story claiming cannabis as a cancer cure generated 4.26 million engagements on social media, while the top accurate news story debunking this false news generated 0.036 million engagements. Cancer organizations infrequently addressed cannabis (average 0.7 Tweets; 0.4 Facebook posts), with low influence compared to false news (average 5.6 versus 527 Twitter retweets; 98 versus 452,050 Facebook engagements, p<0.001).

Conclusions

These findings reveal a growing interest in cannabis use as a cancer cure, and a crucial opportunity for physicians and medical organizations to communicate accurate information about the role of cannabis in cancer to patients, caregivers, and the general public.

## Introduction

Recent claims that cannabis can treat serious health conditions such as cancer have proliferated online, raising concerns within the Food and Drug Administration (FDA) and the oncology community [1]. These claims represent misleading or 'false news' [2], without basis in the medical literature [3]. Although cannabis and its derivatives may help to alleviate disease- and therapy-related symptoms, there is no clinical evidence of its anti-cancer efficacy [4].

The development of this false news is contextualized by three intersecting societal trends: interest in alternative treatments, the proliferation of misinformation online, and the legalization of cannabis. First, the interest in alternative treatments has experienced rapid growth, especially among cancer patients [5]. Viral news and advertisements target cancer patients with inaccurate claims about alternative cancer treatments and may mislead some patients into forgoing conventional therapies [6], potentially resulting in avoidable deaths in patients with curable cancers [7]. Second, there is the increasing use of social media and reliance on the internet for health information, especially on the topic of alternative cancer treatments [8]. Governmental and healthcare organizations [1,9] have tried to curb this spread of misinformation online with limited efficacy [8]. Third, the legalization of cannabis has driven the increased availability of cannabis and an interest in using cannabis as a therapeutic agent beyond the level of scientific evidence [4].

In this study, we characterize the growing online interest in using cannabis as a cancer cure, the relationship between cannabis legalization and online interest, and the role of physicians and leading cancer organizations in clarifying misinformation.

## Materials and methods

Online search activity

Using Google Trends, we characterized the global internet interest in cannabis and cancer from January 2011 through July 2018, as searches for 'cannabis cancer' first gained non-zero relative search volume (RSV) in 2011. Google Trends reports a time-series of RSV for each search term [10]. A search term's monthly RSV is the normalized fraction of all Google searches containing that search term; the normalization constant is chosen such that the maximum RSV achieved across all searched terms is 100. The normalization thus accounts for the effect of increased internet use over time. We compared trends over time for the RSV of 'cannabis cancer' versus the RSV of 'standard cancer therapy' using least squares linear regression (RSV = β0 + β1 * time (month)). 'Standard cancer therapies' queries included 'chemotherapy,' 'radiation therapy,' or 'surgery cancer' by author consensus. For 'cannabis cancer' queries, we reviewed cannabis synonyms suggested by Google Dictionary and Google Trends and included those with a non-zero RSV when combined with the terms 'cancer': 'marijuana', 'cannabidiol (CBD)', 'cannabis', 'tetrahydrocannabinol (THC)', 'weed', or 'cannabinoids'.

Impact of medical and recreational cannabis legalization on online search activity

We determined the association between medical and recreational cannabis legalization and web interest in 'cannabis cancer' over time using the rate of change in RSV in the period between January 2011 and July 2018. We compared the mean rate of change in RSV after normalizing each state to its own highest RSV in the period. States were classified into three legalization groups: states that had legalized medical cannabis before 2011, states that legalized medical cannabis after 2011, and states that never legalized medical cannabis. We also compared the mean rate of change in RSV in states that legalized recreational cannabis after 2011 (no state legalized recreational cannabis before 2011), and states that never legalized recreational cannabis.

Eighteen states did not have enough data on Google Trends, and were excluded from the analysis. Among the remaining 32 states and Washington D.C. with an RSV>0 included in our analysis, 22 states had legalized medical cannabis and seven states had legalized recreational cannabis at the time of our analysis. As Google provides state RSV data that is normalized to the total number of searches within that state, our analyses examined relative changes in RSV over time in each state to control for differing baseline state RSVs. Analysis of covariance (ANCOVA) was used to test between group differences based on their legalization group classification.

News stories with the most social media engagement

We identified the most shared news stories on this topic on social media from July 2017 to July 2018 using Buzzsumo (Buzzsumo, Brighton, England). Buzzsumo is a social media analyzer that evaluates the engagement of news stories, which include likes, comments, and shares on social networks (i.e., Facebook, Twitter, Reddit, and Pinterest) [11-12]. We first performed an analysis using search terms (selected by author consensus) 'cancer' with either 'cure,' 'therapy,' or 'treatment', to find high-impact news stories (those with more than 10,000 engagements) on cancer treatments and to determine what proportion of these news stories discussed cannabis and alternative treatments as cancer treatments. We then used the same 'cannabis cancer' search terms from the Google Trends analysis. High-impact news stories were reviewed independently by the first and last authors. These news stories were classified as accurate news, false news or irrelevant news, based on social media studies on medical misinformation [11,13]. The classification was based on the quality of the scientific information and citation of credible sources; news stories claiming cannabis as a cancer cure were classified as false news. This classification was performed by the first and second authors, with the senior authors adjudicating discrepancies.

Social media activities of leading cancer organizations

Finally, we searched for cannabis and its synonyms in the tweets and Facebook posts published between July 2017 and July 2018 by leading cancer organizations, comprising 27 National Cancer Center Network (NCCN) member institutions and four national organizations: the American Society of Clinical Oncology (ASCO), American Society for Radiation Oncology (ASTRO), National Cancer Institute (NCI) and NCCN. We used the Wilcoxon rank-sum test to compare the number of retweets and the Facebook engagement for these tweets and posts by leading cancer organizations with the Buzzsumo data during the same period for the top false news stories from our social media engagement analysis.

All tests were two-sided with an alpha level of 0.05 unless specified otherwise. Statistical analyses were performed using RStudio (version 1.1.456, R Foundation for Statistical Computing, Vienna, Austria).

## Results

Online search activity of cannabis use in cancer and the impact of legalization

**Figure 1:**
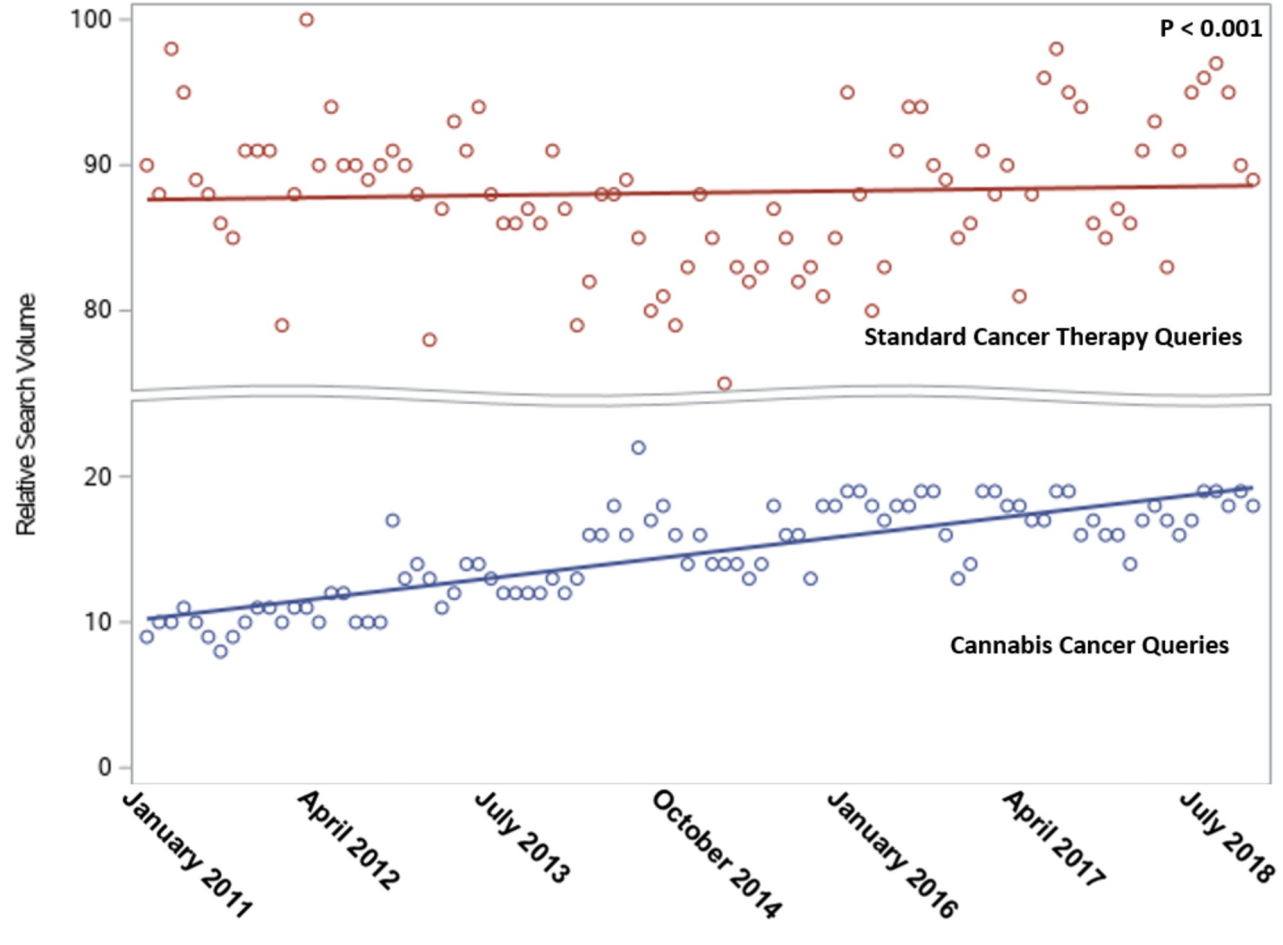
Comparison of relative search volume (RSV) of Google searches for cannabis versus standard therapies for cancer *Monthly RSV is the normalized fraction of all Google searches containing that search term; the normalization constant is chosen such that the maximum RSV achieved across all searched terms is 100. 'Standard cancer therapy' queries included 'chemotherapy', 'radiation therapy', or 'surgery cancer'.

From January 2011 to July 2018, the RSV of 'cannabis cancer' queries increased from 10.3 to 19.3, while the RSV for 'standard cancer therapy' queries changed little, from 87.6 to 88.5 (Figure 1). During the study period, the RSV of 'cannabis cancer' queries increased at a rate 10 times faster than the RSV of 'standard cancer therapies' queries (0.10/month versus 0.01/month, p<0.001). 

**Figure 2:**
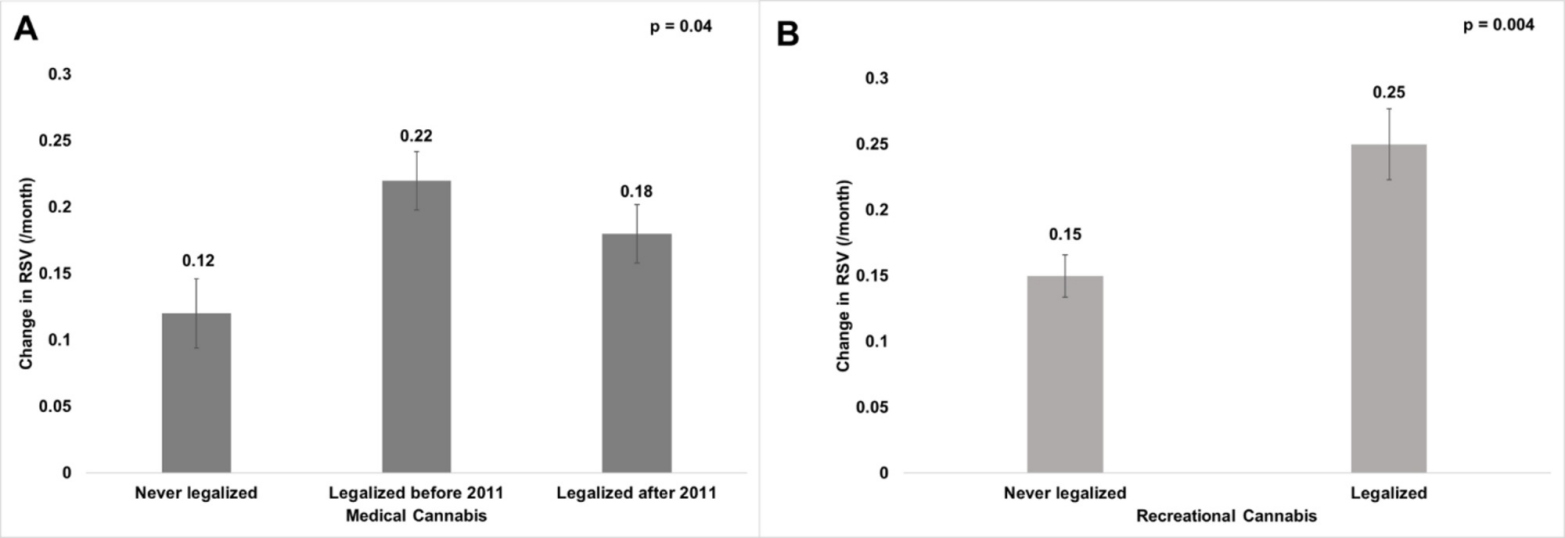
Mean change in state relative search volume (RSV) of cannabis cancer queries during the period of January 2011 – July 2018 by medical cannabis legalization status (A) and by recreational cannabis legalization status (B). *Monthly state RSV is the normalized fraction of all Google searches within a state containing that search term; the normalization constant is chosen such that the maximum RSV achieved across all searched terms is 100.

States that had legalized medical cannabis before 2011 had the highest RSV growth (0.22/month) over the study period, followed by states that had legalized medical cannabis after 2011 (0.18/month); those states that had never legalized medical cannabis had the lowest RSV growth (0,12/month, Figure 2A, p = 0.04). Similarly, states that legalized recreational cannabis showed an RSV growth of 0.25 per month, significantly greater than states that never legalized recreational cannabis (0.15/month, Figure 2B, p=0.004).

Top news stories on cannabis use in cancer on social media

The first social media analysis, which did not specify treatment type, returned 136 high-impact news stories referencing 'cancer cure/therapy/treatment'. Of these, 51 (37.5%) were news stories claiming that alternative treatments cure cancer, 12 (23.5%) of which proposed cannabis as a cancer cure, which was the most common type of alternative cancer treatment represented, more than other alternative treatments such as other plants and physical activities that are claimed to cure cancer. The remaining included 45 (32.4%) news stories on other cancer treatments (chemotherapy, new cancer vaccines, etc.) and 40 (29.4%) irrelevant news stories.

The second social media analysis returned 40 high-impact news stories found on social media referencing 'cannabis cancer.' Of these, 32 (80.0%) were false news that proposed cannabis as cancer cure. Among these, 14 (43.8%) used anecdotes of cancer patients cured by cannabis, while 18 (56.2%) used invalid scientific reasoning. Four (10.0%) were accurate news stories about cannabis as complementary to standard cancer therapies for symptom control. Only one (2.5%) was an accurate news story that debunked the false news that cannabis is a cancer cure ('Pot doesn't cure cancer and stop saying it does, FDA says'). Three (7.5%) were irrelevant news stories (e.g., news about celebrities). Of the ten news stories with the most engagements, eight (80.0%) were false news about cannabis as a cancer cure (Table 1).

**Table 1:** Ten most popular news stories referencing cannabis and cancer on Facebook, Twitter, Reddit and Pinterest ^a ^Engagements include social likes, comments, and shares on social media sites (i.e., Facebook, Twitter, Reddit, and Pinterest). Article covering the same story were combined for engagement.

#	Article Title	URL	Type	Author (type)	Engagements (K)^a^
1	Cancer institute finally admits marijuana kills cancer [14]	https://usahealth24.com/cancer-institute-finally-admits-marijuana-kills-cancer/	News	Website (unknown)	4,267
2	Mum who took illegal cannabis oil to battle terminal cancer given the all-clear [15]	http://www.ladbible.com/community/interesting-mum-who-took-illegal-cannabis-oil-to-battle-terminal-cancer-is-clear-20180612	News	Rebecca Shepherd (reporter)	375.7
3	Cancer survivor tells how cannabis oil 'saved her life' after being told incurable brain tumour would kill her in months [16]	https://www.mirror.co.uk/news/uk-news/cancer-survivor-tells-how-cannabis-11126336	News	Stephanie McCourt (reporter)	329.3
4	Let all terminal cancer patients try cannabis oil [17]	https://you.38degrees.org.uk/petitions/let-all-terminal-cancer-patients-try-cannabis-oil	Campaign	Tracie Lane (unknown)	266.4
5	50-year-old man cures lung cancer with cannabis oil, stuns CBS News [18]	http://www.trueactivist.com/50-year-old-man-cures-lung-cancer-with-cannabis-oil-stuns-cbs-news-t3/	News	Amanda Froelich (unknown)	82.3
6	Study: CBD From marijuana plus chemotherapy tripled cancer survival rates in mice [19]	https://www.forbes.com/sites/daviddisalvo/2018/07/31/study-cbd-from-marijuana-plus-chemotherapy-triples-cancer-survival-rates-in-mice/	News	David DiSalvo (contributor)	78.7
7	There are now 100 scientific studies that prove cannabis cures cancer [20]	https://www.somatagenesis.com/2018/05/12/there-are-now-100-scientific-studies-that-prove-cannabis-cures-cancer/	News	Website (unknown)	45.5
8	Woman rids body of cancer in 4 months using cannabis oil [21]	http://ehealthmagz.com/2018/07/09/woman-rids-body-of-cancer-in-4-months-using-cannabis-oil/	News	Website (unknown)	44.4
9	Pot doesn't cure cancer and stop saying it does, FDA says [22]	https://www.nbcnews.com/health/health-news/pot-doesn-t-cure-cancer-stop-saying-it-does-fda-n816606	News	Maggie Fox (reporter)	36.2
10	Wil Dasovich says medical marijuana helped him battle cancer [23]	http://news.abs-cbn.com/entertainment/02/23/18/wil-dasovich-says-medical-marijuana-helped-him-battle-cancer	News	Website (unknown)	33.8

The top false news story proposing cannabis as a cancer cure, 'Cancer institute finally admits marijuana kills cancer,' generated 4.26 million engagements. Snopes (snopes.com), a fact-checking organization, published a report challenging this false news story [24], but this report generated only 2,207 total engagements. Similarly, the most popular accurate news story debunking the false news ('Pot Doesn’t Cure Cancer and Stop Saying It Does, FDA Says') generated only 36,000 total engagements.

Social media outreach of leading cancer organizations on cannabis use in cancer

**Figure 3:**
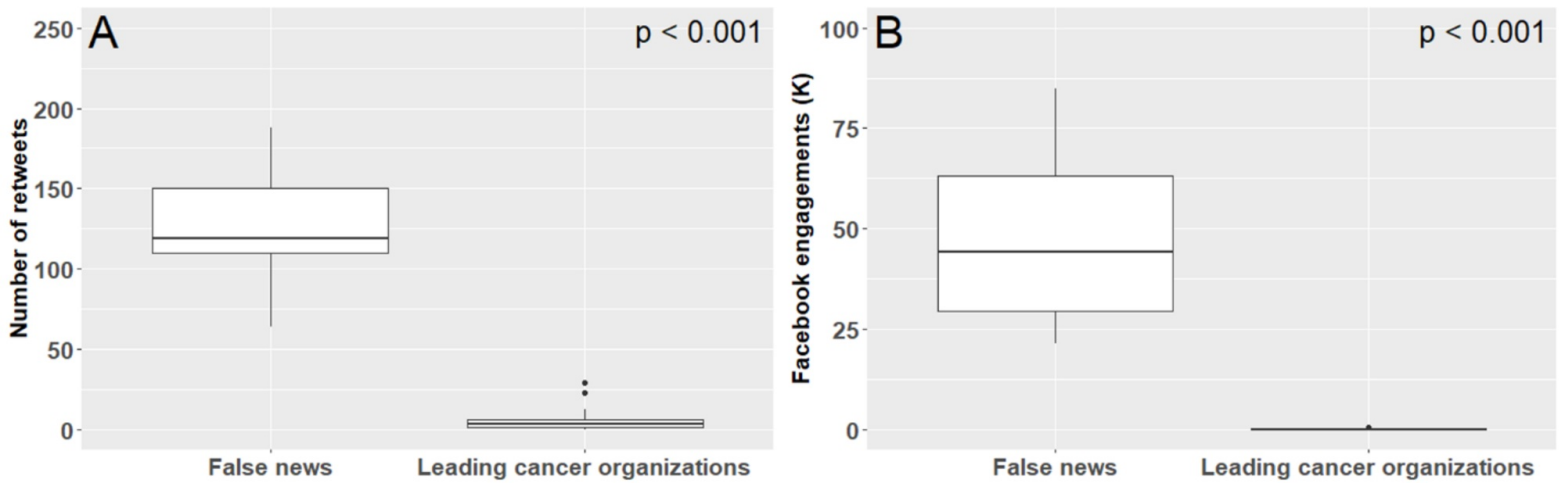
Influence of tweets (A) and Facebook posts (B) by leading cancer organizations compared to that of false news stories (July 2017 – July 2018). Facebook engagement is the sum of likes, shares and comments for each Facebook post.

Of the 31 leading cancer organizations, 30 had Twitter accounts and all had Facebook accounts. From July 2017 to July 2018, the average number of tweets related to cannabis from these accounts was 0.7 (range 0-4), with these tweets having an average of 5.6 (range 0-29) retweets. In comparison, the top 10 false news stories on cannabis and cancer had significantly more retweets (average of 526.6; range 64-3,800; p<0.001; Figure 3A). There were a total of 11 Facebook posts on cannabis made by all leading cancer organizations' Facebook accounts, resulting in an average of 0.4 (range 0-3) posts per account. The average number of Facebook engagements (likes, comments, shares) of these posts was 97.9 (range 0-573). The average number of Facebook engagements of the top ten false news stories that claimed cannabis as a cancer cure was 452,050.1 (range 21,319-3,584,680), significantly more than that of cannabis news stories from leading cancer organizations (p<0.001, Figure 3B). Finally, the National Comprehensive Cancer Care Network (NCCN) member organizations in states where medical or recreational cannabis has not been legalized had no Facebook posts or tweets on cannabis during this period.

## Discussion

Widespread false news of a cannabis cancer cure may be the result of an intersection of three increasing societal trends: interest in alternative treatments, the proliferation of misinformation online, and the legalization of cannabis. In this study, we found a rising online interest in using cannabis for cancer, more so in states that had legalized medical or recreational cannabis. We also found that the most popular news stories shared on social media on this topic misleadingly described cannabis as a cancer cure. In response, leading cancer organizations have rarely addressed the topic of cannabis on social media, and, in these few instances, their outreach has resulted in a markedly low level of influence when compared to false news stories.

The context for these findings is an increasing interest in alternative treatments for a variety of medical conditions. The use of alternative treatments is especially prevalent among cancer patients, increasing from 25% in the 1970s and 1980s to 49% after 2000 [5]. We found that of all the news stories about cancer therapies on social media, 37.5% were news about alternative treatments for cancer; of these news stories, cannabis was the most common alternative treatment claimed to cure cancer. Furthermore, of all the high-impact news stories on cannabis and cancer, 80.0% represented cannabis as a cancer cure, whereas only 2.5% were accurate news stories debunking the use of cannabis as a cancer cure. We also found that online interest in using cannabis as a cancer treatment has nearly doubled in the last seven years (Figure 1). These findings are concerning because patients with cancers treatable by conventional therapies who choose alternative treatments and forgo conventional treatment have a twofold to fivefold increased risk of death [7].

The false news of a cannabis cancer cure is consistent with a broader trend of false news spreading across social media. Use of social media has increased from 5% in 2005 to 69% in 2018 and is now prevalent across all ages and socio-economic groups [25]. Misleading medical news stories may not only cause delays in treatment but may also propagate public distrust in mainstream medicine [3]. Consistent with these findings, we found that among the ten most popular news stories on cannabis and cancer shared on social media, only one debunked the myth of cannabis as a cancer cure (Table 1). Furthermore, the most popular news story, 'Cancer Institute Finally Admits Marijuana Kills Cancer,' had 4.27 million engagements. The most popular news story debunking the myth only generated 0.036 million engagements, less than 1% of the engagements of the one claiming cannabis as a cancer cure.

While false news has a tendency to propagate quickly, widely and deeply [2], the increasing legalization of cannabis – whether for recreational or medical use – is correlated with an increased online interest in cannabis use as a cancer cure (Figure 2). Since the first statewide laws on medical cannabis (California 1996) and recreational cannabis (Colorado 2014) were enacted, the number of Americans with legal and illegal access to cannabis has been steadily growing [26]. It is possible that legalization was a driver of interest and desire for online information about cannabis use in cancer. Studies have shown that the legalization of medical cannabis is associated with a decrease in perceived risk and an increase in the prevalence of cannabis use among patients and non-patients [26]. However, it is also possible that legalization resulted from, rather than led to, rising interest in cannabis for recreational as well as medical use. Whether legalization causally contributes to an interest in a cannabis cancer cure cannot be discerned from the data presented here. However, as more states legalize medical and recreational cannabis and more dispensaries are available, the growing trend of interest in cannabis use in cancer is likely going to continue.

As interest in a cannabis cancer cure is increasing over time – in both states that have legalized its use and in those that have not – the response of the field of oncology is crucial in communicating a more accurate and appropriate framework for cannabis use in cancer care. Although cannabis is shown to alleviate symptoms resulting from cancer and its treatment, it is not a proven treatment or cure of any cancer [4]. The focus of discussions within the oncology community has been on the palliative benefits of cannabis. Indeed, a recent survey showed that 80% of oncologists discuss cannabis with their patients, and 46% recommend medical cannabis for symptom control [27]. However, even for clinicians who endorse the use of medical cannabis for palliative purposes, there may be utility in also discussing its lack of efficacy as a therapeutic option.

While doctor-patient interactions are a crucial venue for the communication of accurate information, many patients who use cannabis may find their primary sources of information outside this relationship. A survey of cannabis users [28] found that 76% reported learning about cannabis from internet research, family members, or friends. In the digital age, where 46% of adults use the internet as their first source of health information, the most trusted sources of online health information – doctors, medical universities, and the federal government [29] – still have the potential to influence public opinions. Given the prominence of online information – particularly for those with cancer [30] – it may be especially important for major oncology organizations to have a robust online engagement policy. However, we found that social media posts by these organizations are minimal and generated significantly less engagement than false news stories (Figure 3). While many of the high-impact news stories on cannabis in cancer use terms such as “CBD” and “CBD oil,” tweets and Facebook posts by leading cancer organizations used only “marijuana” or “cannabis,” potentially failing to engage and influence patients searching for newer cannabis products online. 

The results of this study have important implications for both physicians’ and cancer organizations’ clinical practice and online health communication. With the increasing use of online health information and the lack of reliable metrics to detect misleading medical news [8], there is a clear opportunity for the oncology community to leverage its online influence to debunk misleading medical news.

This study has several limitations. Social media and online search activity around cannabis do not represent the actual use of cannabis. Further, not all states had RSV data available due to periods with low search volume, limiting generalizability. Finally, we could not determine what proportion of the audience of social media news stories about cannabis as a cancer cure were actual patients.

## Conclusions

In conclusion, the false news of a cannabis cancer cure is spreading quickly online and interest in such news stories is rapidly rising. In the face of this concerning increase, there has been a minimal online presence by major cancer hospitals and organizations, representing a crucial opportunity for the oncology community to correct this misinformation and communicate accurate information to patient and caregiver communities.
